# Symbiotic and toxinogenic *Rhizopus* spp. isolated from soils of different papaya producing regions in Mexico

**DOI:** 10.3389/ffunb.2022.893700

**Published:** 2022-10-24

**Authors:** José Francisco Cabrera-Rangel, Judit Valeria Mendoza-Servín, Gonzalo Córdova-López, Raúl Alcalde-Vázquez, Raymundo Saúl García-Estrada, Robert Winkler, Laila P. Partida-Martínez

**Affiliations:** ^1^ Departamento de Ingeniería Genética, Centro de Investigación y de Estudios Avanzados, Irapuato, Mexico; ^2^ Unidad de Genómica Avanzada, Centro de Investigación y de Estudios Avanzados, Irapuato, Mexico; ^3^ Centro de Investigación en Alimentación y Desarrollo, Culiacán, Mexico

**Keywords:** endobacteria, endohyphal bacteria, Rhizopus, Mycetohabitans, rhizoxin, fungal-bacterial Interactions, Mucorales

## Abstract

Mucoralean fungi from the genus *Rhizopus* are common inhabitants of terrestrial ecosystems, being some pathogens of animals and plants. In this study, we analyzed the symbiotic and toxinogenic potential of *Rhizopus* species derived from agricultural soils dedicated to the production of papaya (*Carica papaya* L.) in Mexico. Four representative strains of soil-derived *Rhizopus* spp. were analyzed employing molecular, microscopic, and metabolic methods. The ITS phylogenies identified the fungi as *Rhizopus microsporus* HP499, *Rhizopus delemar* HP475 and HP479, and *Rhizopus homothallicus* HP487. We discovered that *R. microsporus* HP499 and *R. delemar* HP475 harbor similar endofungal bacterial symbionts that belong to the genus *Mycetohabitans* (*Burkholderia* sensu lato) and that none of the four fungi were associated with *Narnavirus* RmNV-20S and RmNV-23S. Intriguingly, the interaction between *R. delemar* - *Mycetohabitans* showed different phenotypes from known *R. microsporus* - *Mycetohabitans* symbioses. Elimination of bacteria in *R. delemar* HP475 did not cause a detrimental effect on fungal growth or asexual reproduction. Moreover, metabolic and molecular analyses confirmed that, unlike symbiotic *R. microsporus* HP499, *R. delemar* HP475 does not produce rhizoxin, one of the best-characterized toxins produced by *Mycetohabitans* spp. The rhizoxin (*rhi*) biosynthetic gene cluster seems absent in this symbiotic bacterium. Our study highlights that the symbioses between *Rhizopus* and *Mycetohabitans* are more diverse than anticipated. Our findings contribute to expanding our understanding of the role bacterial symbionts have in the pathogenicity, biology and evolution of Mucorales.

## Introduction

Mexico is the major exporter of papaya, producing on average 1,117,437.20 metric tons of this tropical fruit per year, harvesting 18,982.79 hectares (SIAP, 2020). Recent studies have shown that *Rhizopus* and other Mucorales are prevalent in papaya producing soils, and some of these fungi, including *Rhizopus delemar*, are also involved in the soft rot of the fruits (Cruz-Lachica et al., 2017; Cruz-Lachica et al., 2018).

Strains of *Rhizopus microsporus* have been reported as the causal agents of rice seedling blight (Furuya et al., 1974), a disease caused by the secreted toxin rhizoxin (Gho et al., 1978; Iwasaki et al., 1984). However, this toxin is not directly synthesized by *R. microsporus*, but by its intracellular bacterial symbionts (Partida-Martinez and Hertweck, 2005). To date, all investigated rhizoxin-producing strains of *Rhizopus microsporus* live in close association with endosymbiotic bacteria from the genus *Mycetohabitans* (*Burkholderia* sensu lato) (Lackner et al., 2009; Partida-Martinez, 2013; Dolatabadi et al., 2016). This novel genus currently recognizes two type species: *M. rhizoxinica* and *M. endofungorum* (Partida-Martinez et al., 2007a; Estrada-de Los Santos et al., 2018). These bacterial symbionts possess the well characterized hybrid PKS-NRPS biosynthetic gene cluster *rhi* for the production of the potent toxin rhizoxin (Partida-Martinez and Hertweck, 2007). This toxin inhibits mitosis in most eukaryotic cells, including plants, fungi, animals and even human cancer cells (Iwasaki et al., 1984; Jordan et al., 1998; Schmitt et al., 2008). The *rhi* biosynthetic gene cluster has been only found and characterized in *Mycetohabitans* spp. and in the plant commensal *Pseudomonas fluorescens* Pf-5 (Brendel et al., 2007). Besides the production of rhizoxin for their fungal host, *Mycetohabitants* symbionts are in full control of the asexual reproduction of *R. microsporus* (Partida-Martinez et al., 2007b) and also influence their sexuality (Mondo et al., 2017). Interestingly, some of these fungi, although apparently less frequently, live also in symbioses with two ssRNA viruses from the genus *Narnavirus* (RmNV-20S and RmNV-23S) (Espino-Vázquez et al., 2020). These narnaviruses, together with *Mycetohabitans*, have been shown to be important for the sexual success in this species (Mondo et al., 2017; Espino-Vázquez et al., 2020). These bacterial and viral symbionts are vertically inherited through the asexual sporangiospores and sexual zygospores produced by *R. microsporus* (Partida-Martinez et al., 2007b; Mondo et al., 2017; Espino-Vázquez et al., 2020).

Here we characterized four representative strains of *Rhizopus* species recovered from papaya producing soils from the states of Colima, Veracruz and Oaxaca in Mexico (Cruz-Lachica et al., 2018). By using molecular, microscopic and metabolic analyses, we identified that two of these strains harbor *Mycetohabitans*, but none of them narnaviruses. Remarkably, we found a novel association between *R. delemar* and *Mycetohabitans* that does not follow the phenotypes known from well-studied and globally distributed *R. microsporus* – *Mycetohabitans* pairs (Lackner et al., 2009; Partida-Martinez, 2013; Dolatabadi et al., 2016). This finding highlights the diversity of the symbioses within these fungal and bacterial genera and expands our possibilities to deepen our understanding of the ecological and evolutionary role of endosymbionts for fungal biology.

## Materials and methods

### Fungal strains and culturing conditions

Wild-type strain *Rhizopus microsporus* HP499, *R. delemar* HP475*, R. delemar* HP479 and *R. homothallicus* HP487 were isolated from soils of papaya producing regions in Colima, Veracruz and Oaxaca in Mexico and provided by García-Estrada in 2019 (Cruz-Lachica et al., 2018). Fungal strains *R. microsporus* ATCC 52813, ATCC 52814 and ATCC 11559 were obtained from the American Type Culture Collection and used as controls for symbiotic/rhizoxin-producing (ATCC 52813 and ATCC 52814) and asymbiotic/non-rhizoxin producing *Rhizopus* species (ATCC 11559). All strains were grown at 30°C in PDA (4 g/L potato extract, 10 g/L dextrose, and 15 g/L agar).

### Isolation and microscopy of bacterial symbionts

Endosymbionts were isolated from their hosts as previously described (Partida-Martinez and Hertweck, 2005; Scherlach et al., 2006; Espino-Vázquez et al., 2020). Briefly, fungal mycelia was submerged on 0.5 mL 0.85% NaCl, mechanically damaged using a pipette tip and then centrifuged for 30 min at 13,200 rpm. Supernatant was plated on TSA petri dishes and incubated at 30°C until the appearance of bacterial colonies. Visualizations of endofungal bacteria were performed using 2-3 days-old grown mycelia (3 cm^2^) on 0.5 mL NaCl 0.85%. An aliquot of 10 µL fungal cell suspension was placed onto a microscope slide then 10 µL of 0.01 mM SYTO™ 9 were added, and the mix was incubated in the dark for 20 min. Fungal tissues were analyzed using a Leica DM600B and the GFP filter.

### DNA isolation and molecular identification of Rhizopus spp. and their endosymbionts

Total DNA from wild-type fungal strains were isolated as described (Nicholson et al., 2001). For fungal molecular identification, the ITS region (Internal Transcribed Spacer) was amplified using ITS-1F (5´- CTTGGTCATTTAGAGGAAGTAA-3´) and ITS-4R (5´- TCCTCCGCTTATTGATATGC-3´) primers. For bacterial molecular identification, the 16S rRNA gene was amplified using 16S-F27 (5´- AGAGTTTGATCMTGGCTCAG-3´) and 16S-R1494 (5´- CTACGGRTACCTTGTTACGAC -3´) primers. *Narnavirus* RmNV-20S and RmNV-23S identification was done as before (Espino-Vázquez et al., 2020). All products were confirmed by Sanger Sequencing at the Genomic Services Laboratory from UGA-Langebio Cinvestav Irapuato, Mexico. Fungal and bacterial sequences have been deposited in Genbank under the following accessions OM677455-458 and OM634667-668, respectively.

### Phylogenetic analyses

The fungal ITS and bacterial 16S rDNA gene sequences were aligned using the MUSCLE algorithm in the MEGA 11 software package (**Table S1**). These alignments were used for tree construction using the Maximum Likelihood method with 1000 bootstrap replicates and the Tamura-Nei model. Also, the same sequences were used to infer their evolutionary history using MrBayes. For this, two independent chains were used along with 100M Monte Carlo Markov chain generations.

### Generation of cured (b-) and re-infected (b*) symbiotic Rhizopus strains

In order to generate cured, symbiont-free fungal strains (b-), the wild-type strains *R. microsporus* HP499 and *R. delemar* HP475 (b+) were constantly cultivated in the presence of ciprofloxacin (50-100 mg/ml) on PDB medium (Partida-Martinez and Hertweck, 2005) until the successful elimination of bacterial symbionts was confirmed by the absence of vegetative sporulation (Partida-Martinez et al., 2007b) and/or the lack of 16S rDNA gene amplification in the antibiotic treated fungi (**Figure S7**). Once cured fungal strains were confirmed and *Mycetohabitans* sp. HP499 and HP475 isolated and validated in axenic culture, co-infections experiments were performed. For this, a mycelial plug of cured fungal strains (b-) was placed on a PDA plate that contained several plugs of 2-days-old cultures of *Mycetohabitans* in TSA medium. Incubation at 30°C took place for ca. 72 hours until the appearance of sporangia was observed.

### Rhizopus’ growth kinetics

All four wild-type (b+ or asymbiotic), bacteria-free (b-) and bacteria-reinfected (b*) Mexican *Rhizopus* strains were cultured on PDA, 30°C in quadruplicate. Radial growth was measured every 24 h. After 96 h, total fungal biomass was dried at 60°C and weighted. Data were analyzed by One-way ANOVA followed by the Tukey’s post-hoc test, P <0.05.

### Fermentation, extraction, bioassays and HPLC-MS analyses for the identification of the toxin rhizoxin

To investigate the production of rhizoxin, liquid fermentations of *Rhizopus* and *Mycetohabitans* species were performed in 100 ml VK medium under agitation at 30°C for 4 days as previously described (Partida-Martinez and Hertweck, 2005). Raw extracts were obtained using 1:1 v/v ethyl acetate. The organic phase was filtered, dried and later dissolved in methanol. Raw extracts were first bioassayed using *Trichoderma atroviridae*, a rhizoxin-susceptible fungus (Schmitt et al., 2008). For this, 2x10^5^ spores of *T. atroviridae* were mixed in 60 ml of PDA in a petri dish. Afterwards, raw extracts of *Rhizopus* spp. (50 µL, 8 mg/mL) and *Mycetohabitans* spp. (50 µL, 2 mg/mL) were poured in the plate. Plates were incubated at 28°C for 3 days and inhibition halos measured (**Figure S8**).

Rhizoxin detection by HPLC-MS was carried out as before (Partida-Martinez and Hertweck, 2005). HPLC analyses were done with the AZXDB-9 Zorbax Eclipse XDB-C18 4.6 x 150 mm column with 5 mm particle size. UV detection was done at 310 nm. HPLC fractions were analyzed by DIESI-MS into a 3D ion trap mass spectrometer LCQ Fleet (Thermo Fisher Scientific). Mass spectra were obtained in ESI positive mode with 4.5 kV, 105 V tube lens voltage, and 30 V capillary voltage at 280°C. The flow injection rate was 10 µL/min. For the MS/MS analysis, main peaks were fragmented with 28 eV collision energy.

### Detection of the rhizoxin biosynthetic cluster (rhi) by PCR

Fragments of five ORFs of the rhizoxin gene cluster (*rhi*) were amplified by PCR using primers designed for *M. rhizoxinica* HKI 454 GenBank: FR687359.1 (**Table S2**). Four of them amplified fragments of the ORFs that build the PKS-NRPS thiotemplate system (rhiA, rhiB, rhiC and rhiD) and one the ORF that codes for the tandem trans-AT (rhiG, **Table S2**) (Partida-Martinez and Hertweck, 2007). The PCR reaction consisted of denaturation for 3 min at 94°C; 30 cycles of 94°C for 30 s, 64°C for 45 s, 72°C for 10 s; and a final extension at 72°C for 30 s. The PCR amplicons were cloned in vector pJET 1.2 (Thermo Fisher Scientific) and sequenced to confirm their identity.

## Results

### Morphological description and taxonomic determination of Rhizopus strains

**Figure 1:**
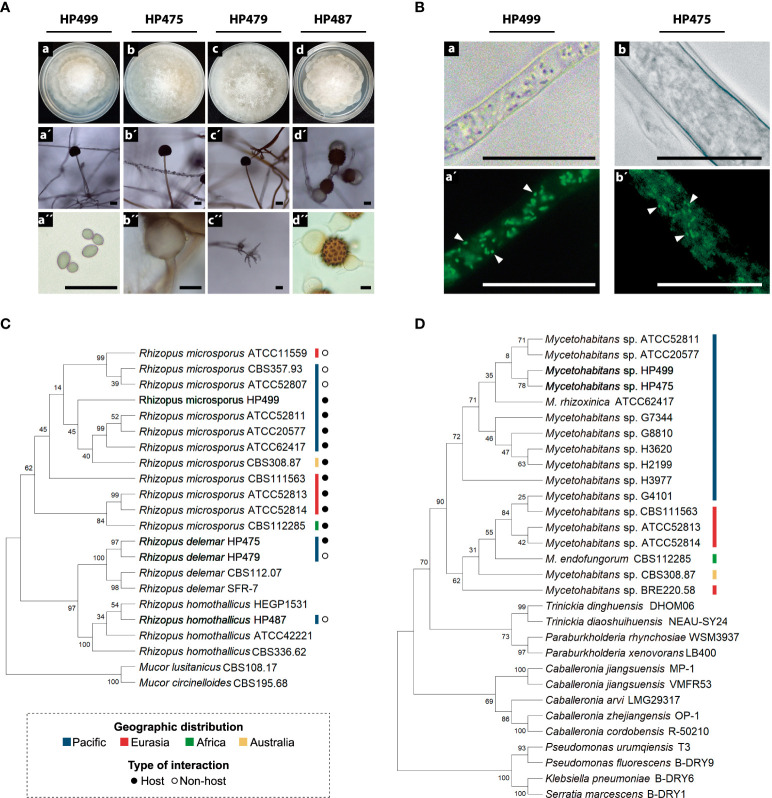
Identification of *Rhizopus* spp. and their bacterial symbionts. **(A)** Morphological structures of *Rhizopus* spp. **(a-d)** Colonies grown on PDA at 30°C for 72 h. **(a´-c´)** Sporangium and sporangiophore. **(d´)** and **(d´´)** Zygospores. **(a´´)** Sporangiospores. **(b´´)** Columella. **(c´´)** Rhizoids. Scale bars = 20 μm. **(B)** Bacterial endosymbionts thriving in the cytoplasm of the fungal strains HP499 and HP475. DNA from bacterial symbionts was stained with SYTO™ 9. **(a-b)** Micrographs taken under white light, and **(a´-b´)** under fluorescence. Arrows point out individual bacterial cells. Scale bars = 20 μm. **(C)** Fungal phylogeny based on the ITS marker. **(D)** Bacterial phylogeny based on the 16S rRNA gene. The evolutionary history was inferred for **(C, D)** by using the Maximum Likelihood method with 1000 bootstrap replicates and Tamura-Nei model.

Morphological observations during the vegetative development of the *Rhizopus* strains showed the characteristic rhizoids, grayish branched aerial mycelium, and elliptic shape spores (**Figure 1A**) (Schipper and Stalpers, 1984). According to the ITS phylogenetic analyses (**Figures 1C**, **S3**), the identities of the four Mexican fungal strains are *R. microsporus* HP499, *R. delemar* HP475, *R. delemar* HP479, and *R. homothallicus* HP487 for which sexual reproductive structures were observed as this fungus in not heterothallic (**Figure 1Ad´, d´´**) (Gryganskyi et al., 2018).

### Identification of bacterial and viral endosymbionts

We detected positive 16S rRNA gene amplification in *R. microsporus* HP499 and *R. delemar* HP475 (**Figure S1**). Thus, we searched and confirmed the presence of bacteria inside the fungal cytosol by SYTO™ 9 staining (**Figures 1B** and **S2**). Phylogenetic analyses grouped these bacteria closer to *M. rhizoxinica* ATCC 62417 and into the Pacific branch (**Figures 1D**, **S4**). Accordingly, we named these bacteria as *Mycetohabitans* sp. HP499 and *Mycetohabitans* sp. HP475. We also succeeded in isolating and cultivating both bacterial symbionts that showed to be gram-negative small bacilli (**Figure S5**). Additionally, we investigated the presence of the *Narnavirus* RmNV-20S and RmNV-23S by RT-PCR. However, none of the four fungal Mexican strains harbored these viruses (**Figure S6**).

### The absence of Mycetohabitans alters R. microsporus HP499 phenotype, but not that of R. delemar HP475

**Figure 2:**
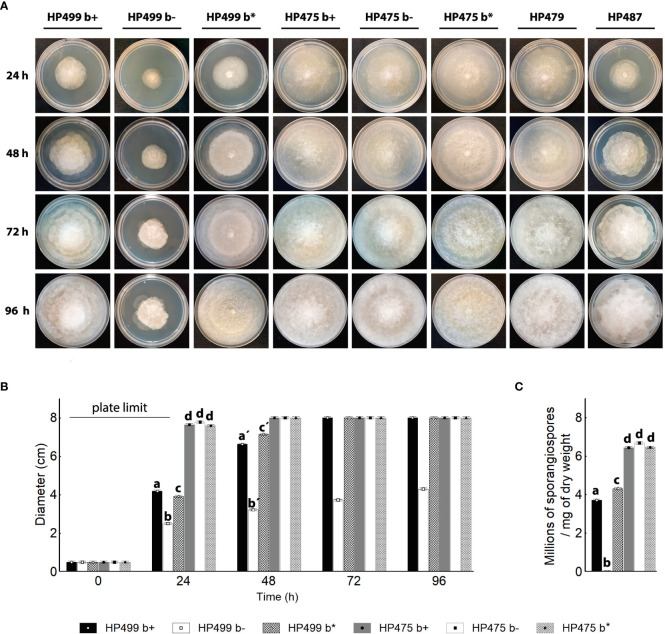
Development of symbiotic and asymbiotic *Rhizopus* strains. **(A)** Development (up to 96 h) of colonies of *Rhizopus microsporus* HP499, *R. delemar* HP475 and HP479, and *R. homothallicus* HP487 grown on PDA at 30°C. b+ natural symbiotic strain. b- cured strain. b* re-infected strain with their natural bacteria. **(B)** Growth kinetics of wild-type, cured, and re-infected fungal hosts for 96 h on PDA plates. Error bars represent the standard error. One-way ANOVA followed by Tukey’s post-hoc test, P <0.05, n = 4 in each group. Letters indicate statistical significance. **(C)** Sporangiospores produced per mg of dry weight after 96h of growth. One-way ANOVA followed by Tukey’s post-hoc test, P <0.05, n = 4. Error bars represent the standard error.

To address the importance of the endofungal bacteria in *R. microsporus* HP499 and *R. delemar* HP475, we generated symbiont-free (b-) and re-infected fungi (b*) (**Figure S7**). *R. microsporus* HP499 significantly decreased its growth in the absence of its *Mycetohabitans* endobacteria, being the wild-type (wt) phenotype recovered when the fungus was re-infected. Remarkably, none of the manipulated strains of *R. delemar* HP475 (b-, b*) changed its growth rate with respect to the wild-type (wt) fungus throughout 96h (**Figures 2A, B**). The asymbiotic *R. delemar* HP479 and *R. homothallicus* HP487 showed similar growth rates as the symbiotic *R. delemar* HP475 and *R. microsporus* HP499, respectively (**Figure 2A**). Encouraged by the strong effect of *Mycetohabitans* sp. HP499 on the growth of its host, we searched for changes in the asexual reproduction of the fungus (Partida-Martinez et al., 2007b). As expected, absence of the endofungal bacteria nullifies the production of sporangiospores in *R. microsporus* HP499, however, in *R. delemar* HP475 no changes in the number of sporangiospores produced were quantified in the absence of the bacterial symbionts (**Figure 2C**).

### Mycetohabitans sp. HP475 does not produce the potent toxin rhizoxin

**Figure 3:**
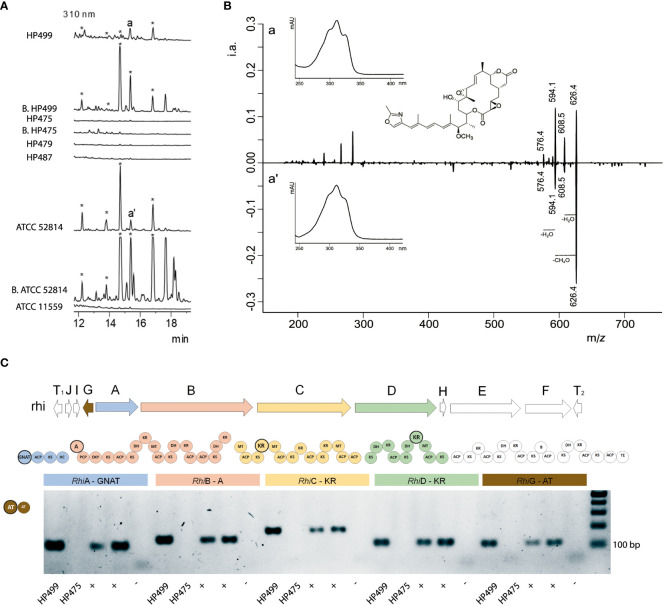
Metabolic and molecular identification of the production of rhizoxin. **(A)** HPLC chromatograms of raw extracts obtained from the liquid fermentation of *Rhizopus* and *Mycetohabitans* species. *R. delemar* HP479, *R. homathallicus* HP487 and *R. microsporus* ATCC 11559 are asymbiotic fungal strains. *R. microsporus* HP499, *R. delemar* HP475 and *R. microsporus* ATCC 52814 are symbiotic fungal strains, and *Mycetohabitans* sp. HP499 [B. HP499], *Mycetohabitans* sp. HP475 [B. HP475]) and *Mycetohabitans* sp. ATCC 52814 [B. ATCC 52814] are their respective endofungal bacteria. Strains ATCC 52814 and ATCC 52814 [B. ATCC 52814] were used as positive, and ATCC 11559 as negative controls, respectively. Peaks labeled with a, a’, and * represent rhizoxin derivatives. **(B)** Characteristic spectra of rhizoxin (Maximum at 310 nm) and its MS/MS (626 m/z) fragmentation pattern in HP499 (a) and ATCC 52814 (a’) strains. Collision energy was 28 eV. **(C)** PCR amplification of the *rhi* cluster (rhzoxin). *R. microsporus* HP499 does have gene fragments from the rhizoxin cluster, while *R. delemar* HP475 does not show any. Reference fungal strains ATCC 52813 and ATCC 52814 harboring *Mycetohabitans* were used as positive controls (positives for rhizoxin production), while asymbiotic strain ATCC 11559 served as negative control. Highlighted circles represent the amplified motifs within the *rhi* BGC. GNAT *N*-acetyltransferase, ACP acyl carrier protein, KS ketosynthase, HC condensation/heterocyclization, A adenylation, PCP peptidyl carrier protein, OXY oxygenase, DH dehydratase, KR ketoreductase, MT *C*-methyltransferase, B unknown domain possibly involved in β-branching, TE thioesterase, RhiA-F thiotemplate, RhiI *O*-methyltransferase, RhiG acyltransferase, RhiH cytochrome P450 monooxygenase, and T_1_, T_2_ transposase genes.

We evaluated the capacity to produce rhizoxin and its derivatives by the four Mexican *Rhizopus* strains, as well as by the two new isolated bacterial symbionts *Mycetohabitans* sp. HP499 and HP475. First, we evaluated the antifungal capacity of raw extracts vs. *Trichoderma atroviridae*, a rhizoxin-susceptible fungus (Schmitt et al., 2008). This bioassay showed that only the raw extracts of *R. microsporus* HP499 and its symbiotic bacterium *Mycetohabitans* sp. HP499 inhibited the growth of *T. atroviridae* (**Figure S8**). Our HPLC-MS analyses confirmed that *R. microsporus* HP499 and *Mycetohabitans* sp. HP499 did produce rhizoxin and its derivatives (**Figures 3A, B**). These rhizoxin-like peaks were MS/MS analyzed and compared to those produced by the well characterized rhizoxin-producing strain *R. microsporus* ATCC 52814 wt (**Figure S9**), confirming the same chemical nature. However, symbiotic *R. delemar* HP475 nor its *Mycetohabitans* bacteria inhibited the growth of *T. atroviridae* nor produced rhizoxin (**Figures 3A**, **S8**), while asymbiotic *R. delemar* HP479 and *R. homothallicus* HP487 did not inhibit growth of *T. atroviridae* and did not produce rhizoxins, as expected.

Finally, we investigated if the lack of rhizoxin and its derivatives in symbiotic *R. delemar* HP475 and its endobacteria was due to the absence of the *rhi* cluster. As depicted in **Figure 3C**, fragments of the *rhiG*, *rhiA*, *rhiB*, *rhiC* and *rhiD* ORFs were readily amplified from the genomic DNA from *R. microsporus* HP499 and the rhizoxin-positive *R. microsporus* ATCC 52813 and 52814, but not from *R. delemar* HP475.

## Discussion

Papaya is a highly valued agricultural product of Mexico. In this study, we investigated the symbiotic and toxinogenic potential of four *Rhizopus* strains that were recovered from soils from the states of Colima, Veracruz and Oaxaca in which papaya is produced (Cruz-Lachica et al., 2018). Our results revealed that two of these strains harbor bacterial symbionts from the genus *Mycetohabitans*, but none of them the narnaviruses RmNV-20S and RmNV-23S. These symbiotic fungal strains were identified as *R. microsporus* HP499 and *R. delemar* HP475 (**Figure 1C**). Microscopic observations and molecular identification of these bacterial symbionts showed that they thrive in the fungal cytoplasm (**Figure 1B**), are most similar to each other, and close to the type strain *Mycetohabitans rhizoxinica* ATCC 62417 that belongs to the Pacific clade (**Figure 1D**). Significantly, in this Pacific clade are now strains from Japan, USA and Mexico (**Figures 1C, D**; **S3** and **S4**; **Table S1**). These results support the biogeographical distribution of symbiotic fungi and their endobacteria (Lackner et al., 2009). Notably, our report that *R. delemar* HP475 harbor *Mycetohabitans* symbionts is, as far as we are aware, unique. Other recent studies searching for endofungal bacteria in the phylum Mucoromycota, or specifically in the genus *Rhizopus*, have revealed that *Mycetohabitans* is the most common symbiont of *Rhizopus microsporus* (Okrasińska et al., 2021). In fact, only until recently, all *Mycetohabitans* strains known were associated with several strains of this fungal species (Lackner et al., 2009; Partida-Martinez, 2013; Dolatabadi et al., 2016), as the origin of *Mycetohabitans* species obtained from clinical specimens could not be clearly tracked (Gee et al., 2011). Recently, novel *Mycetohabitans* symbionts living in four different strains of *Mortierella verticillata* have been discovered, although their capacity to produce rhizoxin has not been reported nor their effects on the biology of *Mortierella* fungi (Büttner et al., 2021). Lastly, a clinical isolate of *R. microsporus* stably associates with *Ralstonia pickettii*. This bacterial symbiont helps its fungal host to evade amoeba and cause opportunistic infections in animals (Itabangi et al., 2022). All these reports and our evidence support the notion that interactions of *Rhizopus* species with endofungal bacteria are more diverse and plastic than initially thought.

Moreover, our findings that the *Mycetohabitans* symbiont of *R. delemar* HP475 does not influence fungal growth nor is in control of its asexual sporulation (**Figure 2**) suggest that this symbiont might be of more recent acquisition. Horizontal transfer of *Mycetohabitans* symbionts in *R. microsporus* has been postulated as possible for the following reasons: a) *Mycetohabitans* spp. can readily colonize cured *R. microsporus* hosts (Partida-Martinez and Hertweck, 2005), as they possess a functional type 2 secretion system that enables the release of chitinolytic enzymes, specially chitinase, that allow bacterial entry into the fungal hyphae (Moebius et al., 2014); b) Mucoralean fungi tend to be rhizoxin-resistant, as their amino acid in the 100 position of their β-tubulin is not arginine (N), but serine (S) or alanine (A) (Schmitt et al., 2008). These changes in this amino acid prevent β-tubulin binding with rhizoxin, and further allows microtubule polymerization during mitosis; and c) Phylogenies of symbiotic *Rhizopus microsporus* and their *Mycetohabitans* symbionts revealed a high degree of co-speciation, but also suggested the possibility of occasional horizontal transfers (Lackner et al., 2009). All these make us hypothesize that *R. delemar* HP475 might have acquired its *Mycetohabitans* symbionts by contact with symbiotic *R. microsporus* in the papaya producing soils sampled. It is also likely that the horizontal transfer of *Mycetohabitans* symbionts to a different species of *Rhizopus* could have imposed genomic re-arrangements in the endobacteria to adapt to the new host. One such adaptation could possibly be the full or partial loss of the *rhi* cluster. This hypothesis warrants further and deeper investigation.

In addition, our experiments and evidence to date have not revealed yet any further contribution of *Mycetohabitans* to the fitness/adaptation of *R. delemar* HP475. However, we cannot discard that *Mycetohabitans* sp. HP475 could confer other ecological advantages to its fungal host in its natural environment, as members from this genus have the highest metabolic potential in the genus *Burkholderia* sensu lato, despite their relatively reduced genomes (Mullins and Mahenthiralingam, 2021). This has been shown for *Mycetohabitans* sp. CBS 308.87 (**Figure 1D**; **Figure S4** and **Table S1**), a symbiotic bacterium of *R. microsporus* that produces low amounts of rhizoxin and its derivatives, but which has the biosynthetic gene cluster *nec* to produce cytotoxic benzolactones called necroximes A, B, C and D (Niehs et al., 2020). Remarkably, the *nec* BGC was also found in the endofungal bacterial symbiont *Candidatus* Mycoavidus necroximicus that thrives inside *M. verticillata* NRRL 6337 and produces necroximes C and D. These metabolites protect its fungal host from nematode attacks, increasing fungal survival in the soil (Büttner et al., 2021).

From the agricultural perspective, our study points out to the necessity of studying the prevalence of symbiotic and toxinogenic *Rhizopus* species and other Mucorales in Mexico to prevent agricultural losses, and also the consumption of toxic fruits that could promote disease in humans and animals.

In sum, the two novel symbiotic relationships identified here will help expand our understanding of the distribution, ecology and evolution of fungal-bacterial-viral symbioses in early-diverging fungi.

## Data availability statement

The datasets presented in this study can be found in online repositories. The names of the repository/repositories and accession number(s) can be found in the article/**Supplementary Material**.

## Author contributions

JC-R, JM-S & LP-M: Planned and designed research. JC-R, JM-S, GC-L & RA-V: Performed experiments and analyzed the data. RG-E: Collected soil samples and isolated *Rhizopus* spp. RA-V, RW & LP-M: Generated and analyzed MSn data. LPP-M: Secured funding. JC-R, JM-S, GC-L, RA-V & LP-M: Wrote the paper. All authors contributed to the article and approved the submitted version.

## Funding

LP-M acknowledges Consejo Nacional de Ciencia y Tecnología in Mexico (CONACyT), which supported this project with grants: FOINS-2015-01-006 and A1-S-9889.

## Acknowledgments

We are thankful to María Nélida Vázquez Sánchez, Laboratorio de Cromatografía/Dra. Danae Carrillo, Laboratorio de Microscopía/Dr. Lino Sánchez, and Antonio Cisneros, all from Cinvestav Unidad Irapuato, for their technical support and help with chromatography, microscopy and photography, respectively.

## Conflict of interest

The authors declare that the research was conducted in the absence of any commercial or financial relationships that could be construed as a potential conflict of interest.

## Publisher’s note

All claims expressed in this article are solely those of the authors and do not necessarily represent those of their affiliated organizations, or those of the publisher, the editors and the reviewers. Any product that may be evaluated in this article, or claim that may be made by its manufacturer, is not guaranteed or endorsed by the publisher.
