# Supplementary figures and images for: Symbiotic and toxinogenic Rhizopus spp. isolated from soils of different papaya producing regions in Mexico
Source: Front Fungal Biol. 2022 Oct 24;3:893700. doi: 10.3389/ffunb.2022.893700 (PMC10512248; doi:10.3389/ffunb.2022.893700)

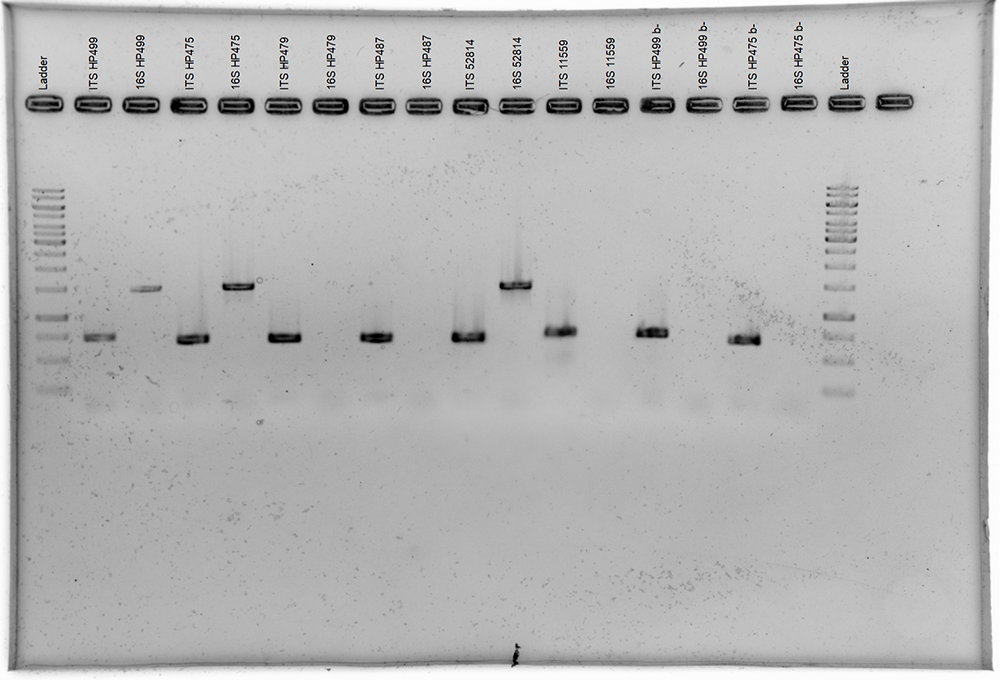

Supplement: Supplementary file 1 [file DataSheet_1.zip › Supplementary Material/FS1_ITS_16s.tif]

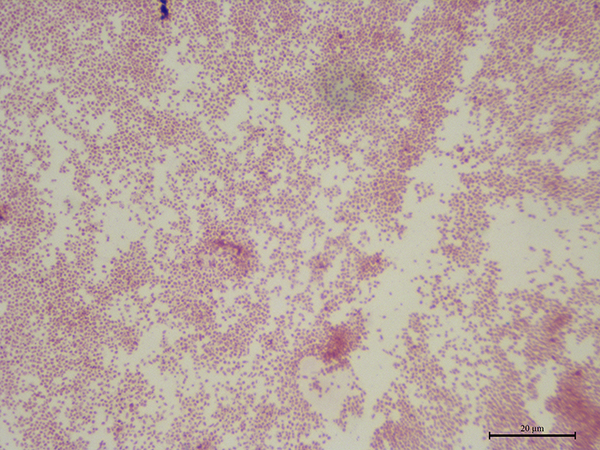

Supplement: Supplementary file 1 [file DataSheet_1.zip › Supplementary Material/FS2_B4_ATCC52813.tif]

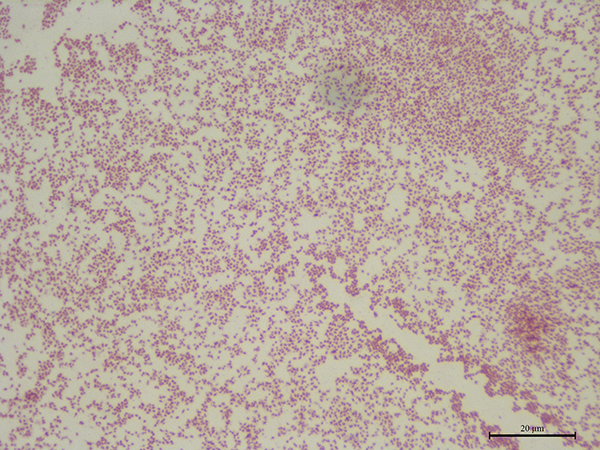

Supplement: Supplementary file 1 [file DataSheet_1.zip › Supplementary Material/FS2_BHP475.tif]

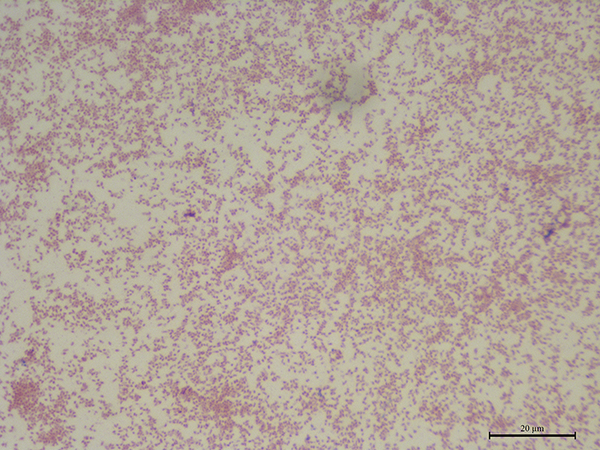

Supplement: Supplementary file 1 [file DataSheet_1.zip › Supplementary Material/FS2_HP499.tif]

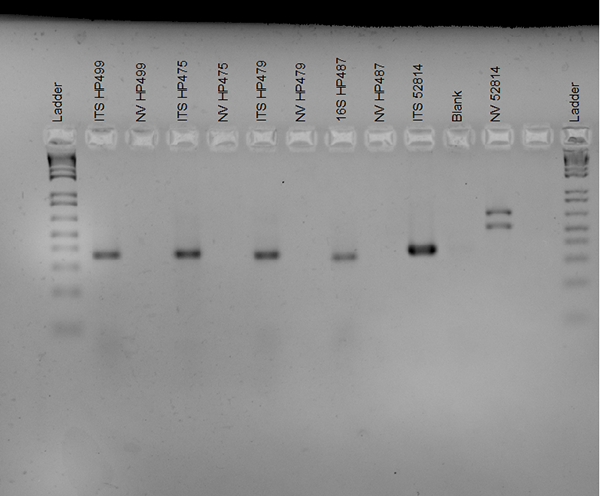

Supplement: Supplementary file 1 [file DataSheet_1.zip › Supplementary Material/FS3_Narnaviruses.tif]

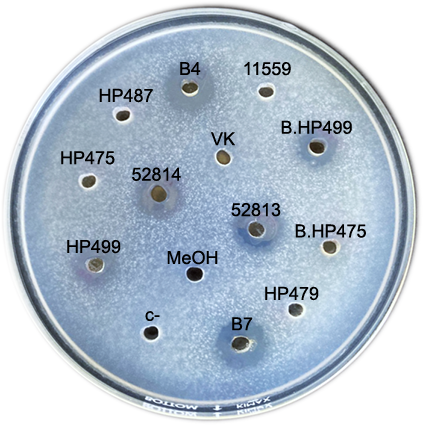

Supplement: Supplementary file 1 [file DataSheet_1.zip › Supplementary Material/FS4_Antibiosis.tif]
