# Supplementary material for: Symbiotic and toxinogenic Rhizopus spp. isolated from soils of different papaya producing regions in Mexico
Source: Front Fungal Biol. 2022 Oct 24;3:893700. doi: 10.3389/ffunb.2022.893700 (PMC10512248; doi:10.3389/ffunb.2022.893700)
Supplement: Supplementary file 2 [file DataSheet_2.pdf]

Supplementary Material

Symbiotic and Toxinogenic *Rhizopus* spp. Isolated from Soils of Different Papaya Producing Regions in Mexico

J. Francisco Cabrera-Rangel†, J. Valeria Mendoza-Servín†, Gonzalo Córdova-López, Raúl Alcalde-Vázquez, Raymundo Saúl García-Estrada, Robert Winkler and Laila P. Partida-Martínez*

† These authors have contributed equally to this work and share first authorship.

***Correspondence:** Laila P. Partida-Martínez (laila.partida@cinvestav.mx)

Index of Supplementary Figures and Tables

Table S1. List of the ITS and 16S rRNA gene sequences used for the phylogenies included in Figure 1, S3 and S4.

Table S2. Primers used for the amplification of the *rhi* cluster based on the sequenced and annotated genome of *M. rhizoxinica* HKI 454.

Figure S1. Molecular genotyping of Mexican strains of *Rhizopus* spp. Amplification by PCR of the fungal genetic marker ITS and the bacterial genetic marker 16s rRNA from total gDNA.

Figure S2. Fungal strains *R. delemar* HP479 and *R. homothallicus* HP487 do not harbor bacterial endosymbionts.

Figure S3. Phylogenetic tree of selected fungal ITS sequences.

Figure S4. Phylogenetic tree of selected bacterial 16S rRNA gene sequences.

Figure S5. Isolated and cultivated endofungal bacterial strains derived from *R. microsporus* HP499 and *R. delemar* HP475.

Figure S6. Evaluation of the presence of *Narnavirus* RmNV-20S and *Narnavirus* RmNV-23S in the four Mexican fungal strains.

Figure S7. Molecular genotyping of *R. microsporus* HP499 and *R. delemar* HP475 with their wild-type (b+), cured (b-) and re-infected (b*) bacterial endosymbionts.

Figure S8. Evaluation of the antifungal activity produced by raw extracts of *Rhizopus* spp. and their endofungal bacteria on *Trichoderma atroviride* growing on PDA and incubated at 28 °C for 3 days.

Figure S9. Rhizoxin-like Ms/Ms fragmentation patterns of HPLC fractions marked with * in Figure 3A at retention time 12.1, 13.7, 14.5-14.6 and 15.1-15.3 min, respectively.

Table S1. List of the ITS and 16S rRNA gene sequences used for the phylogenies included in Figure 1, S3 and S4.

**Fungal ITS**					
Strain ID	NCBI Reference Sequence / GenBank	Origin	References	Harbor bacterial symbionts?	Comments
Rhizopus microsporus ATCC11559	AY243959.1	USSR	This work	No	CBS 344.29
Rhizopus microsporus CBS357.93	AB097392	Tempeh, Indonesia	Unpublished	No	Identified as R. azygosporus in 2002
Rhizopus microsporus ATCC52807	MH861534.1	Bread, China	10.1016/j.simyco.2018.05.001	No	CBS631.82
***Rhizopus microsporus* HP499**	OM677455.1	Soils of papaya producing regions in Colima, Veracruz and Oaxaca in Mexico	This work and 10.1016/j.funbio.2018.04.008	Yes, *Mycetohabitans* sp. HP499	Previously identified as *R. microsporus* HP499 based on the 28S, partial sequence (KX980509).
Rhizopus microsporus ATCC52811	KC206539	Not specified, USA	10.1007/s13225-013-0229-6	Yes, *Mycetohabitans* sp. ATCC52811	CBS261.28
Rhizopus microsporus ATCC20577	FN182225.1	Soil, Japan	10.1128/AEM.01765-08	Yes, *Mycetohabitans* sp. ATCC20577	F-1360<br>HKI 414
Rhizopus microsporus ATCC62417	FN182224.1	Rice seedlings, Japan	10.1128/AEM.01765-08	Yes, *Mycetohabitans* sp. ATCC62417	HKI 413<br>*R. chinensis* Rh-2
Rhizopus microsporus CBS308.87	AY803933.1	Man, from deep necrotic tissue within the hand following a spider bite, Australia	10.1016/j.jdermsci.2005.01.010	Yes, *Mycetohabitans* sp. CBS308.87	HKI 415
Rhizopus microsporus ATCC111563	AY243959.1	Sufu starter: rice wine tablet, Vietnam	10.1007/s13225-013-0229-6	*Yes,* *Mycetohabitans* sp. ATCC111563	HKI 399
Rhizopus microsporus ATCC52813	AB097385.1	Soil, Ukraine	10.1007/s13225-013-0229-6	Yes, *Mycetohabitans* sp. ATCC52813	CBS699.68

Rhizopus microsporus ATCC52814	AB097386	Forest soil, Georgia	10.1007/s13225-013-0229-6	Yes, *Mycetohabitans* sp. ATCC52814	CBS700.68
Rhizopus microsporus ATCC112285	KC206520.1	Ground nuts, Mozambique	10.1007/s13225-013-0229-6	Yes, *Mycetohabitans* sp. ATCC112285	HKI 383<br>MRC 303
***Rhizopus delemar* HP475**	OM677456.1	Soils of papaya producing regions in Colima, Veracruz and Oaxaca in Mexico	This work and 10.1016/j.funbio.2018.04.008	Yes, Mycetohabitans sp. HP475	Previously identified as *R. oryzae* HP475 based on the 28S, partial sequence (KX980497)
***Rhizopus delemar* HP479**	OM677457.1	Soils of papaya producing regions in Colima, Veracruz and Oaxaca in Mexico	This work and 10.1016/j.funbio.2018.04.008	No	Previously identified as *R. stolonifer* HP479 based on the 28S, partial sequence (KX980504)
Rhizopus delemar CBS112.07	NR_103595.1		10.1093/database/bau061	No	*Rhizopus oryzae* CBS112.07
Rhizopus delemar SFR-7	MT540020.1		Unpublished		*Rhizopus arrhizus* SFR-7
Rhizopus homothallicus HEGP1531	KU926333.1		Unpublished		
***Rhizopus homothallicus* HP487**	OM677458.1	Soils of papaya producing regions in Colima, Veracruz and Oaxaca in Mexico	This work and 10.1016/j.funbio.2018.04.008		Previously identified as *R. homothallicus* HP487 based on the 28S, partial sequence (KX980502)
Rhizopus homothallicus ATCC42221	AF543525.1		Unpublished		
Rhizopus homothallicus CBS336.62	NR_103616.1		10.1093/database/bau061		
Mucor lusitanicus CBS108.17	NR_126127.1		10.1371/journal.ppat.1002086		
Mucor circinelloides CBS195.68	NR_126116.1		10.1128/JCM.44.2.340-349.2006		

Bacterial 16S rRNA gene					
Strain ID	NCBI Reference Sequence / GenBank	Origin	References	Fungal Host	Other strain IDs
Caballeronia arvi LMG29317	NR_145594.1	Soil	10.3389/fmicb.2016.00877		
Caballeronia cordobensis R-50210	NR_133710.1	Agricultural soil	10.1099/ijs.0.059667-0		
Caballeronia jiangsuensis MP-1	NR_133991.1	Methyl parathion contaminated soil	10.1099/ijs.0.064444-0		
Caballeronia jiangsuensis VMFR53	MZ234637.1				
Caballeronia zhejiangensis OP-1	NR_117902.1	wastewater-treatment system	10.1099/ijs.0.035428-0		
Klebsiella pneumoniae B-DRY6	MG996452.1	food samples	10.1007/s00203-021-02251-4	*Rhizopus* sp.	
Mycetohabitans endofungorum CBS 112285	NR_042584.1		10.1099/ijs.0.64660-0<br>10.1128/AEM.01765-08	*Rhizopus microsporus* CBS 112285	HKI 456<br>B5
Mycetohabitans rhizoxinica ATCC62417	NR_042393.1		10.1099/ijs.0.64660-0<br>10.1038/nature03997<br>10.1128/AEM.01765-08	*Rhizopus microsporus* ATCC62417	HKI 454<br>B1
Mycetohabitans sp. BRE220.58	MZ330685.1		10.1073/pnas.2110669118	*Mortierella verticillata* NRRL 6337	
Mycetohabitans sp. G4101	HQ005412.1	Clinical Specimens	10.1371/journal.pone.0015731		
Mycetohabitans sp. G7344	HQ005405.1	Clinical Specimens	10.1371/journal.pone.0015731		
Mycetohabitans sp. G8810	HQ005406.1	Clinical Specimens	10.1371/journal.pone.0015731		
Mycetohabitans sp. H2199	HQ005408.1	Clinical Specimens	10.1371/journal.pone.0015731		
Mycetohabitans sp. H3620	HQ005410.1	Clinical Specimens	10.1371/journal.pone.0015731		
Mycetohabitans sp. H3977	HQ005411.1	Clinical Specimens	10.1371/journal.pone.0015731		
Mycetohabitans sp. ATCC52813	AJ938144.1	*Rhizopus microsporus*	10.1128/AEM.01765-08	*Rhizopus microsporus* ATCC52813	HKI 402<br>B4<br>*Mycetohabitans* sp. B13

Mycetohabitans sp. ATCC52814	FN186054	*Rhizopus microsporu s*	10.1128/AEM. 01765-08	*Rhizopus microsporus* ATCC52814	HKI 403<br>B7<br>*Mycetohabitans* sp. B14
Mycetohabitans sp. CBS 308.87	AJ938143.1	*Rhizopus microsporu s*	10.1128/AEM. 01765-08	*Rhizopus microsporus* CBS 308.87	HKI 404<br>B8
Mycetohabitans sp. CBS 111563	FN186052	*Rhizopus microsporu s*	10.1128/AEM. 01765-08	*Rhizopus microsporus* CBS 111563	HKI 455<br>B3
Mycetohabitans sp. ATCC 20557	AJ938141.1	*Rhizopus microsporu s*	10.1128/AEM. 01765-08	*Rhizopus microsporus* ATCC 20557	HKI 512<br>B2
Mycetohabitans sp. ATCC52811	FN186053	*Rhizopus microsporu s*	10.1128/AEM. 01765-08	*Rhizopus microsporus* ATCC52811	HKI 513<br>B6
***Mycetohabitans* sp. HP475**	OM634668.1	*Rhizopus delemar*	This work	*Rhizopus delemar* HP475	
***Mycetohabitans* sp. HP499**	OM634667.1	*Rhizopus microsporu s*	This work	*Rhizopus microsporus* HP499	
Paraburkholderia rhynchosiae WSM3937	NR_116248.1	*Rhynchosia ferulifolia* root nodules	10.1099/ijs.0.0 48751-0		
Paraburkholderia xenovorans LB400	NR_074325.2	PCB-contaminat ed site	10.1073/pnas. 0606924103		
Pseudomonas fluorescens B-DRY9	MG996521.1	food sources	10.1007/s0020 3-021-02251-4	*Rhizopus* sp.	
Pseudomonas urumqiensis T3	NR_171524.1	Rhizospher e soil of *Alhagi sparsifolia*	10.1099/ijsem. 0.003390		
Serratia marcescens B-DRY1	MG996443.1	food sources	10.1007/s0020 3-021-02251-4	*Rhizopus delemar*	
Trinickia diaoshuihuensis NEAU-SY24	NR_171504.1	Soil	10.1099/ijsem. 0.003155		
Trinickia dinghuensis DHOM06	NR_171482.1	Forest soil	10.1099/ijsem. 0.003324		

Table S2. Primers used for the amplification of the *rhi* cluster based on the sequenced and annotated genome of *M. rhizoxinica* HKI 454 (Partida-Martinez and Hertweck, 2007).

Primers ID	Sequence	™ (°C)	Expected amplicon size	Gene amplified	CDS Region in nucleotide sequence
RhiA-F	ATTACGATCTGGCCAGCCG	64	124 bp	GNAT family N-acetyltransferase	NC_014722.1 1647978-1655060 (-)
RhiA-R	TCCGATTGACCCAAGCACC	64.3			
RhiB-F	TTGGCGGATCATCTCGTCG	63.8	153 bp	Amino acid adenylation domain-containing protein	NC_014722.1 1627380-1647785 (-)
RhiB-R	GAAGCACTGCGCATCATCG	63.6			
RhiC-F	TTGGCCATTGTGAAAGCGC	64.2	199 bp	SDR family NAD(P)-dependent oxidoreductase	NC_014722.1 1611611-1627318 (-)
RhiC-R	GACGAAATCGCCGCAATCC	63.6			
RhiD-F	GAATCGTTTGGCGCGATCC	63.6	122 bp	SDR family NAD(P)-dependent oxidoreductase	NC_014722.1 1599081-1611560 (-)
RhiD-R	ACACTGGTCGCCATCTTGG	64.2			
RhiG-F	ATCTCCTGCCACAACACCG	64.2	112 bp	ACP S-malonyltransferase	NC_014722.1 1655795-1657792 (+)
RhiG-R	CTTCTCGTTCAATCCGCGC	63.2			

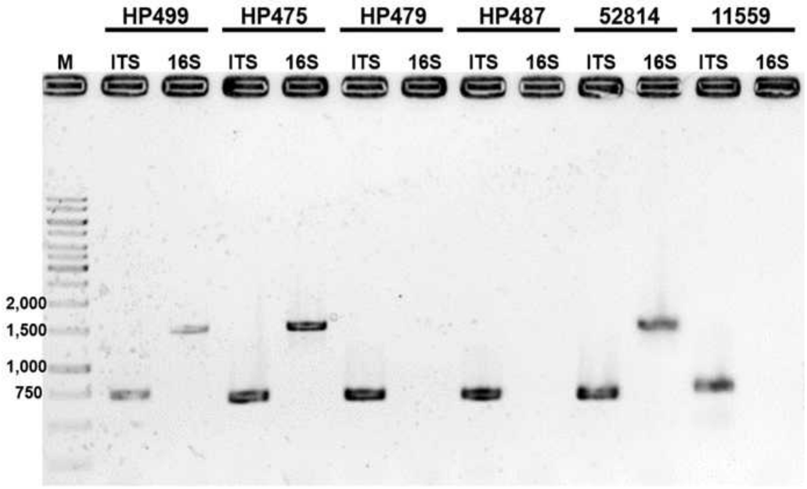

Figure S1. Molecular genotyping of Mexican strains of *Rhizopus* spp. Amplification by PCR of the fungal genetic marker ITS and the bacterial genetic marker 16s rRNA from total gDNA. The strains *R. microsporus* ATCC 52814 and ATCC 11559 were used as positive and negative controls, respectively.

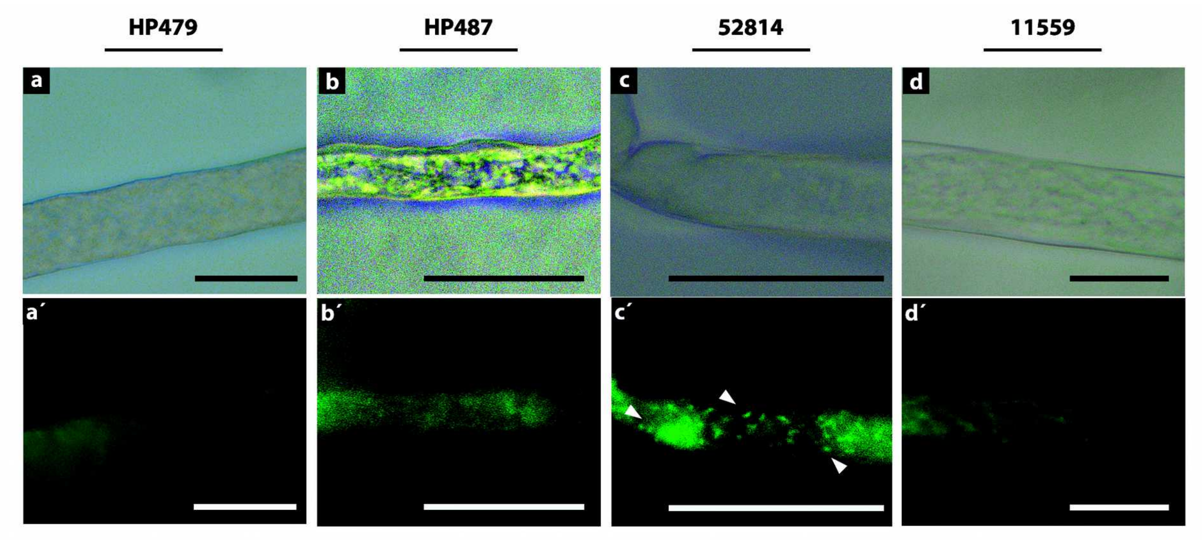

Figure S2. Fungal strains *R. delemar* HP479 and *R. homothallicus* HP487 do not harbor bacterial endosymbionts. Cells were stained with SYTO™ 9. a-d Micrographs taken under white light, and a´-d´ under fluorescence. The strains *R. microsporus* ATCC 52814 and ATCC 11559 were used as positive and negative controls, respectively. Arrows point out individual bacterial cells. Scale bars = 20 μm.

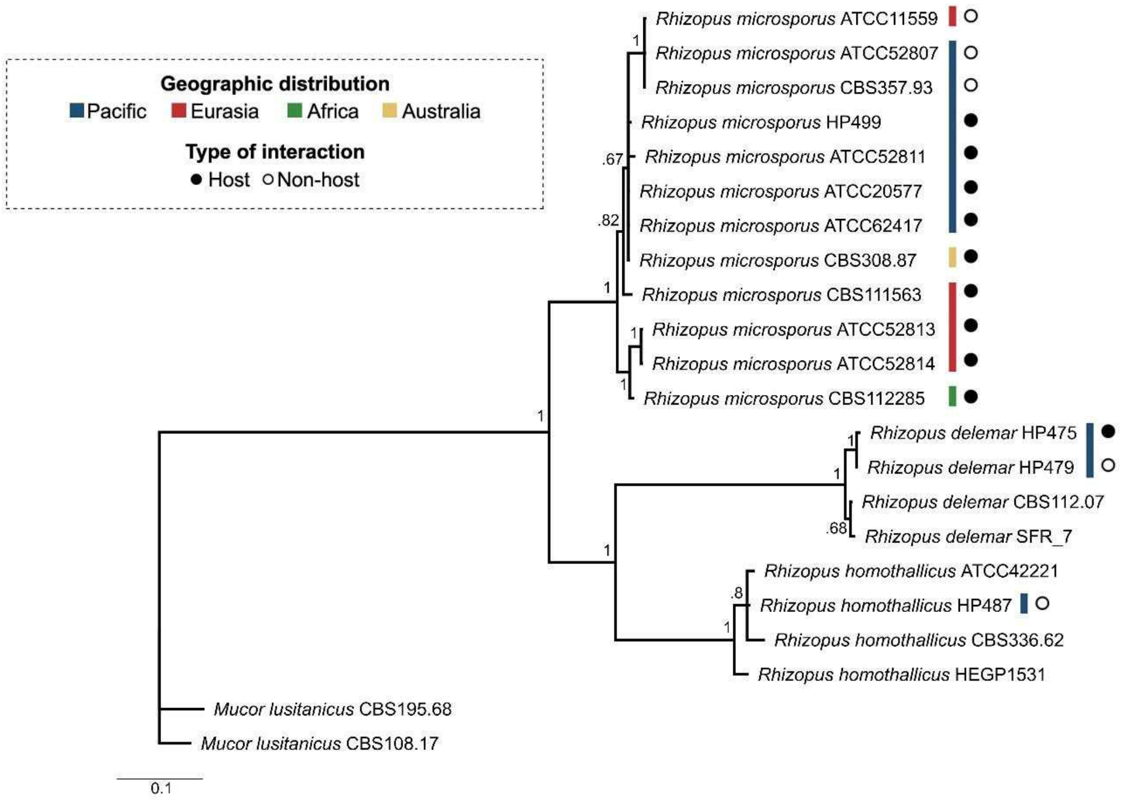

Figure S3. Phylogenetic tree of selected fungal ITS sequences. The evolutionary history was inferred using MrBayes. Two independent chains were used along with 100M Monte Carlo Markov chain generations. Probability support is shown next to each node. *Mucor circinelloides* CBS195.68 was used as the outgroup.

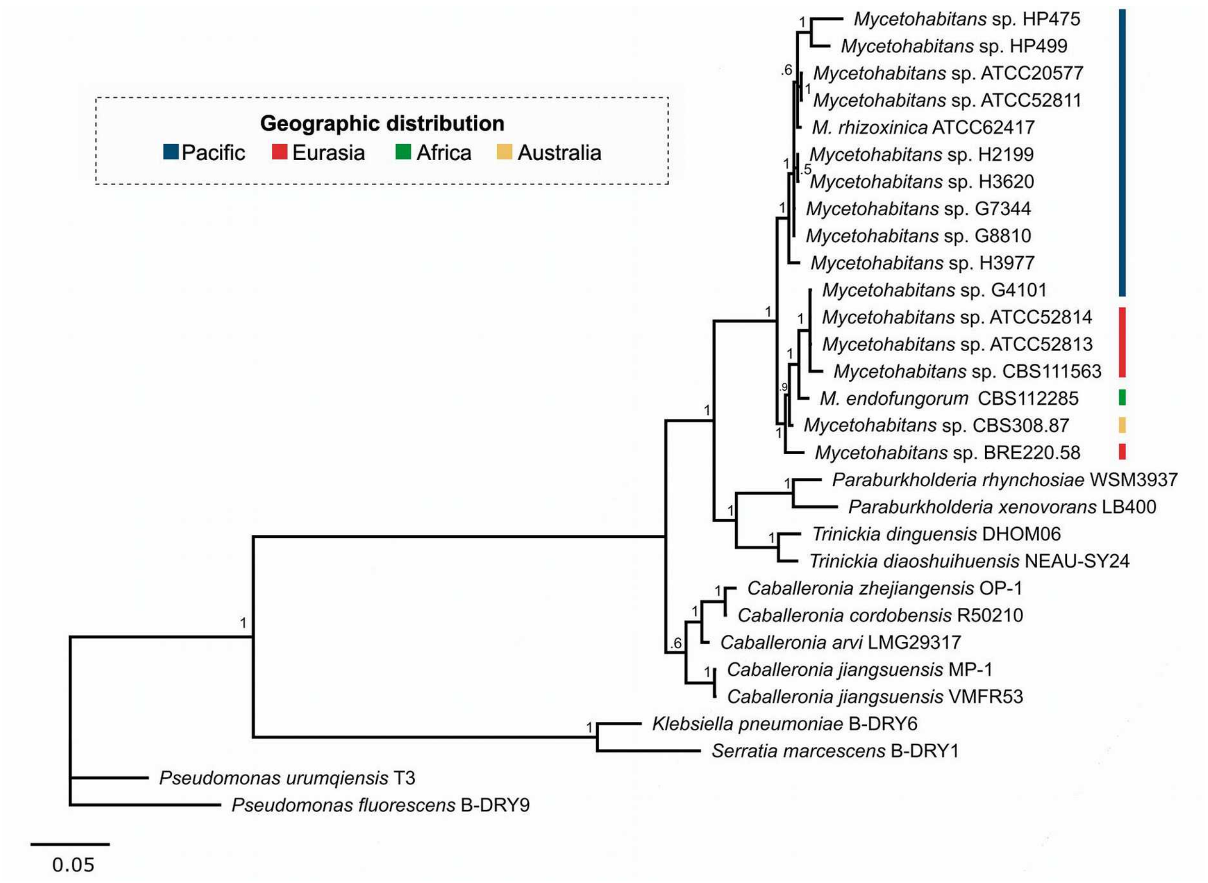

Figure S4. Phylogenetic tree of selected bacterial 16S rRNA gene sequences. The evolutionary history was inferred using MrBayes. Two independent chains were used along with 100M Monte Carlo Markov chain generations. Probability support is shown next to each node. *Pseudomonas urumqiensis* T3 was used as the outgroup.

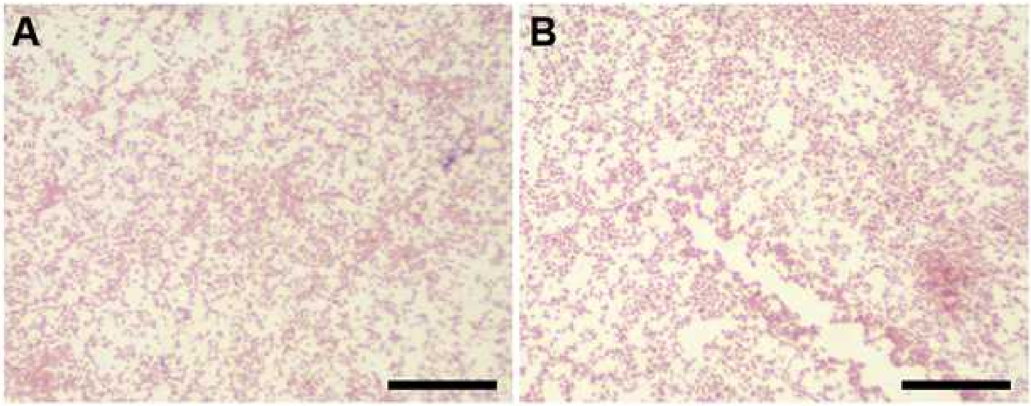

Figure S5. Isolated and cultivated endofungal bacterial strains derived from *R. microsporus* HP499 (**A**) and *R. delemar* HP475 (**B**). In both cases, bacilli and gram-negative bacteria are observed. Scale bars = 20 µm.

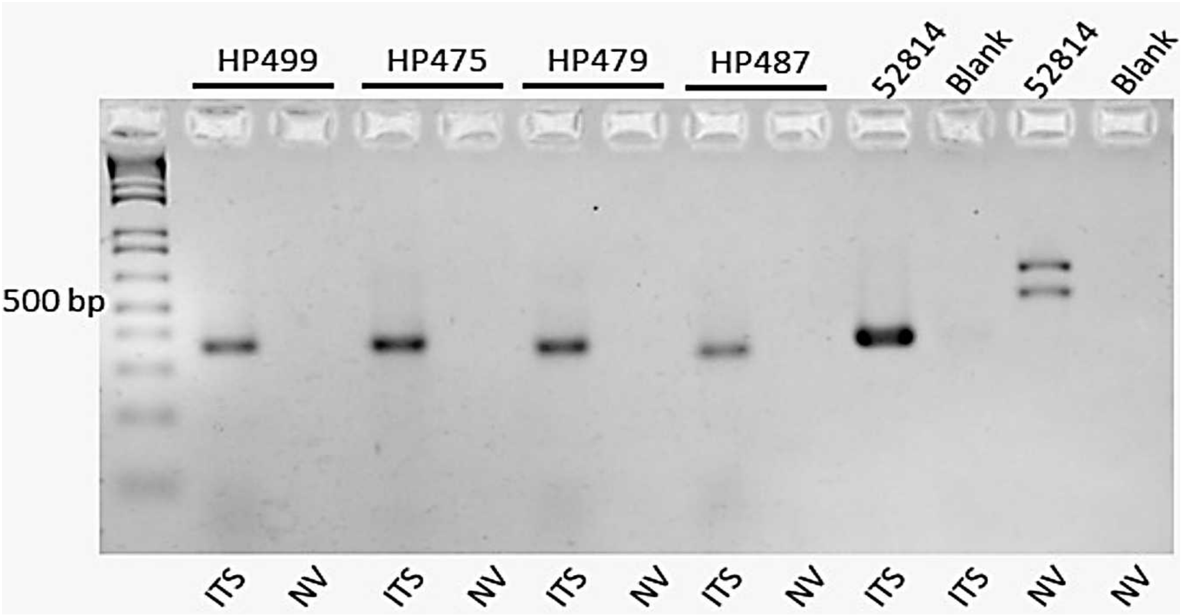

Figure S6. Evaluation of the presence of *Narnavirus* RmNV-20S and *Narnavirus* RmNV-23S in the four Mexican fungal strains. Amplification by RT-PCR of genome fragments of the RmNV-20s (683 pb) and RmNV-23s (537pb) narnaviruses. The strain *R. microsporus* ATCC 52814 was used as positive control (Espino-Vazquez *et al.*, 2020).

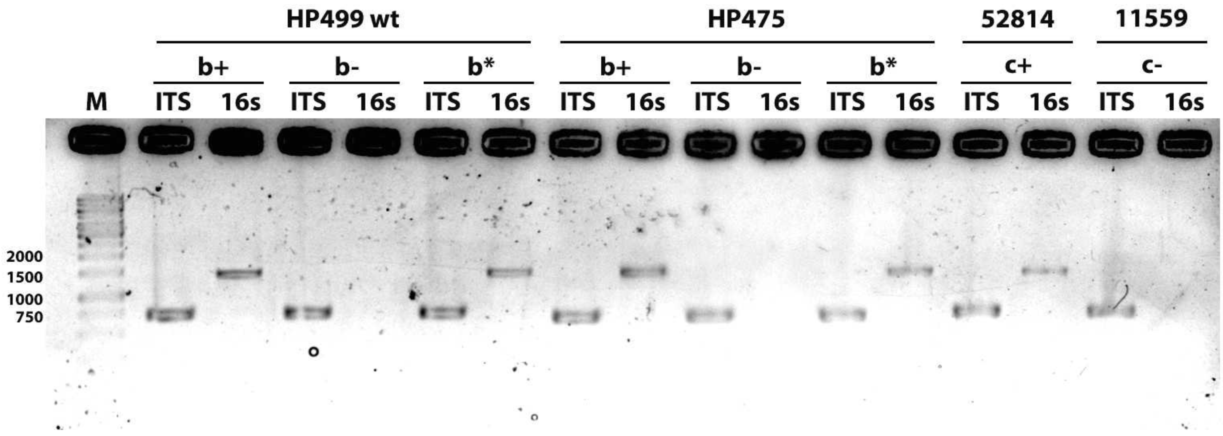

Figure S7. Molecular genotyping of *R. microsporus* HP499 and *R. delemar* HP475 with their wild-type (b+), cured (b-) and re-infected (b*) bacterial endosymbionts. The strains *R. microsporus* ATCC 52814 and ATCC 11559 were used as positive and negative controls, respectively.

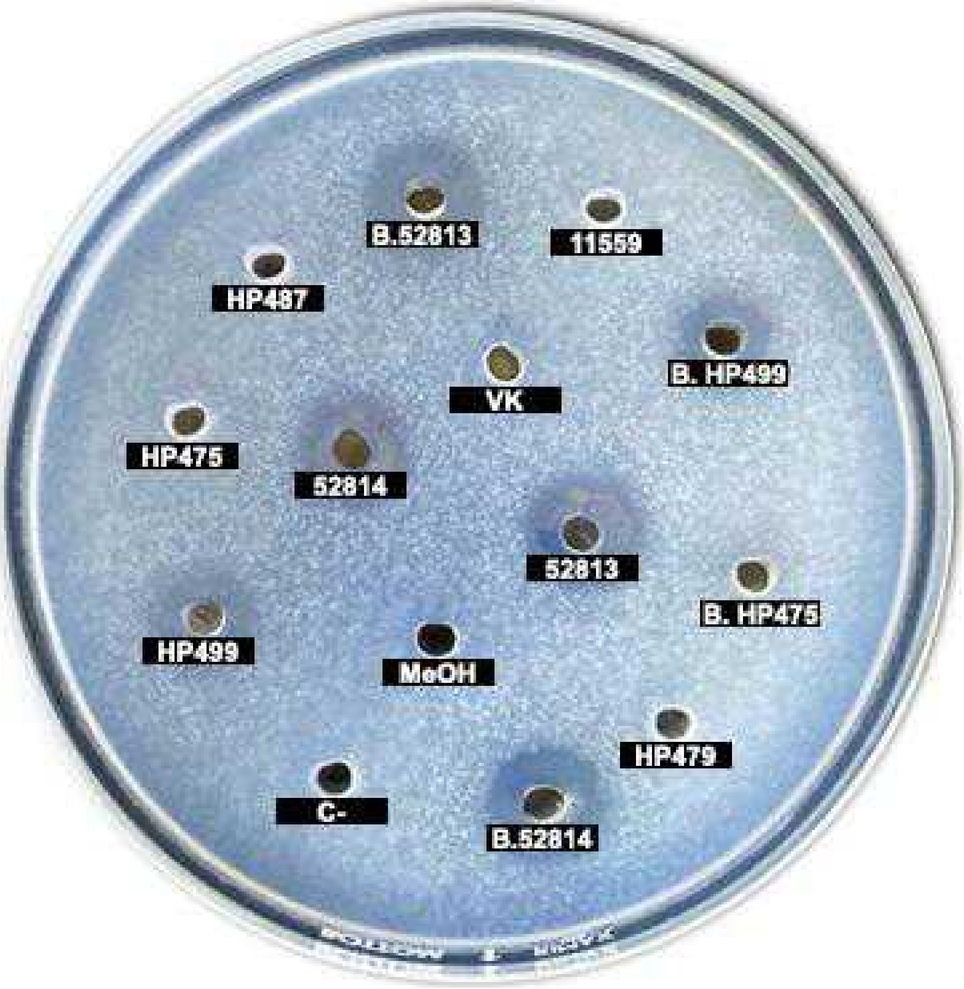

Figure S8. Evaluation of the antifungal activity produced by raw extracts of *Rhizopus* spp. and their endofungal bacteria on *Trichoderma atroviride* growing on PDA and incubated at 28 °C for 3 days. Fungal and bacterial raw extracts were produced after axenic fermentations in VK medium for the following strains: *R. microsporu*s HP499, ATCC 52813, ATCC 52814 and ATCC 11559, *R. delemar* HP475 and HP479, *R. homothallicus* HP487, *Mycetohabitans* sp. ATCC 52813 [B.52813], *Mycetohabitans* sp. ATCC 52814 [B.52814], *Mycetohabitans* sp. HP499 [B. HP499] and *Mycetohabitans* sp. HP475 [B. HP475]). The fungi *R. microsporus* ATCC 52813 and ATCC 52814, and the bacterial strains *Mycetohabitans* sp. ATCC 52813 and *Mycetohabitans* sp. ATCC 52814 were used as positive controls. The strain *R. microsporus* ATCC 11559, pure methanol (MeOH) and the VK medium (c-) were used as negative controls.

MS/MS in *R. microsporus* HP499	MS/MS in *R. microsporus* ATCC 52814
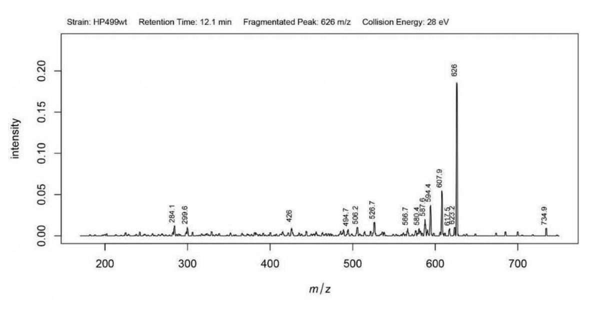	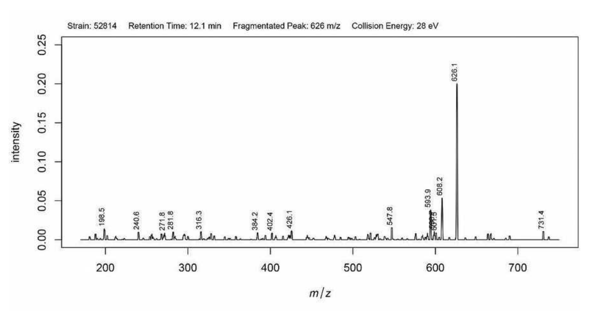
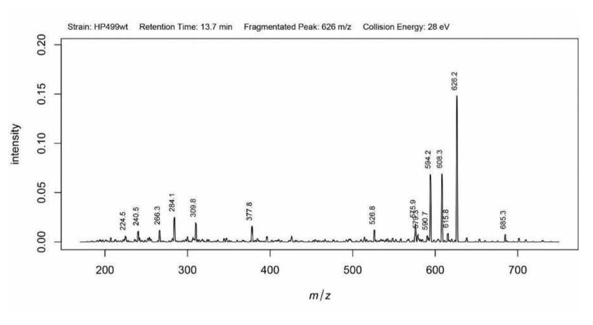	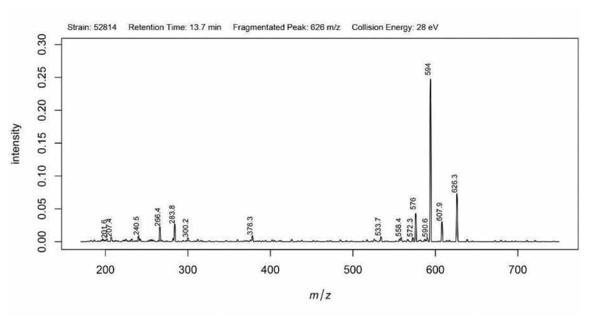
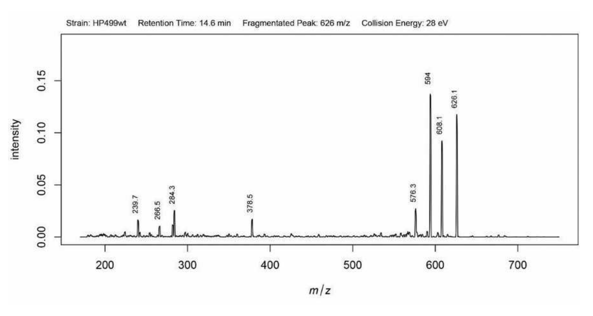	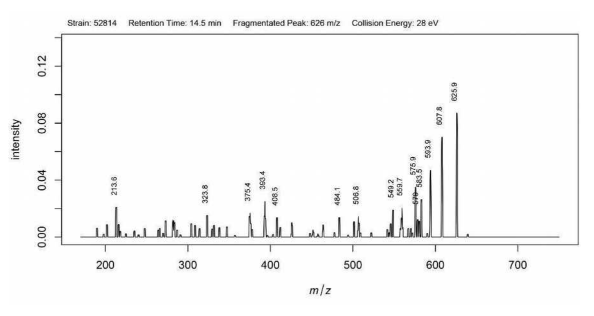
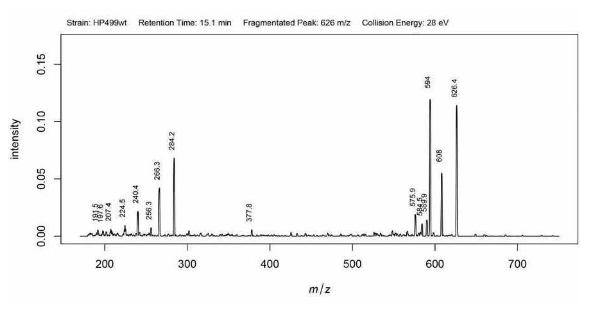	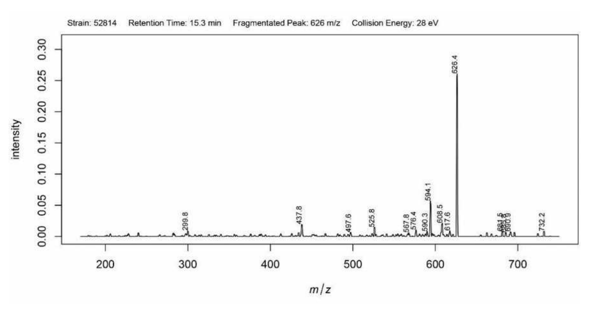

Figure S9. Rhizoxin-like Ms/Ms fragmentation patterns of HPLC fractions marked with * in Figure 3A at retention time 12.1, 13.7, 14.5-14.6 and 15.1-15.3 min, respectively.

References

Espino-Vázquez, A. N., Bermúdez-Barrientos, J. R., Cabrera-Rangel, J. F., Córdova-López, G., Cardoso-Martínez, F., Martínez-Vázquez, A., et al. (2020). Narnaviruses: novel players in fungal-bacterial symbioses. *ISME J.* 14, 1743–1754. doi:10.1038/s41396-020-0638-y.

Partida-Martinez, L. P., and Hertweck, C. (2007). A Gene Cluster Encoding Rhizoxin Biosynthesis in "*Burkholderia rhizoxina*", the Bacterial Endosymbiont of the Fungus *Rhizopus microsporus*. *ChemBioChem* 8, 41–45. doi:10.1002/cbic.200600393.
